# Synthesis of an Efficient S/N-Based Flame Retardant and Its Application in Polycarbonate

**DOI:** 10.3390/polym10040441

**Published:** 2018-04-14

**Authors:** Weiqiu Wen, Jianwei Guo, Xi Zhao, Xiong Li, Hongmei Yang, Jem-Kun Chen

**Affiliations:** 1 School of Chemical Engineering & Light Industry, Guangdong University of Technology, Guangzhou 510006, China; 15024028760@163.com (W.W.); zhaoxizel@outlook.com (X.Z.); lixong@163.com (X.L.); yanghongmei2003@163.com (H.Y.); 2 Department of Materials Science and Engineering, National Taiwan University of Science and Technology, 43, Sec. 4, Keelung Road, Taipei 106, Taiwan

**Keywords:** flame retardant, adamantane, polycarbonate, fire properties, water-resistance

## Abstract

Considering the poor compatibility and water-resistance of sulfonate flame retardants for polycarbonate (PC), an efficient S/N-based flame retardant named 1,3,5,7-tetrakis(phenyl-4-sulfonyl-melamine)adamantane (ASN) has been developed. Fire properties studies of PC/ASN blends indicate that the addition of 0.10 wt % ASN imparts a V-0 rating and a limited oxygen index (LOI) value of 30.1% to PC specimens, and ASN can suppress the heat and toxic gas release of PC composites. Additionally, PC/ASN blends are believed to be exceptional materials for outdoor PC applications due to their superior water-resistance properties. Moreover, mechanical properties were further systematically investigated, and the correlative results indicate that the tensile strength and rigidity of specimens are improved with the addition of ASN.

## 1. Introduction

Polycarbonate (PC) is a desirable material in many applications, and is commonly used in the fields of automotive, construction, and electronics due to its outstanding merits such as high glass transition temperature, good thermostability, and excellent electrical properties [1,2]. Although PC is born with a V-2 rating and a limited oxygen index (LOI) value of approximately 25.0% [3], more stringent flame retardancy of PC is frequently required in certain applications (e.g., electronics, construction) due to safety concerns. Therefore, reducing the flammability of PC composites is still a challenging and rewarding subject [4,5,6,7].

To reduce the combustibility of PC, incorporating it with additive-type flame retardants (FRs) is generally an effective approach. Traditionally, halogen-based FRs have been broadly developed and utilized to enhance the flame retardancy of PC. However, the applications of halogen-based FRs have been restrained for their adverse effects on the environment and public health [8,9,10]. Consequently, an increasing requirement for the development of halogen-free FRs has been brought forward. In recent years, halogen-free FRs have been widely developed and applied (e.g., phosphorus-based [11,12,13,14], silicon-based [15,16,17], nitrogen-based [18], boron-based [19], and sulfur-based). According to some research, sulfur-based FRs are believed to be more effective for flame-retarding PC than other FRs [20,21,22]. Indeed, PC composites incorporated with quite a small amount (less than 1 wt %) of sulfur-based FRs can present satisfactory flame retardance. For example, the commercially-available potassium diphenylsulfone sulfonate (KSS) can show a V-0 rating and an LOI value of 37% with 0.25 wt % addition [23]. In our previous work, 1,3,5,7-tetrakis(phenyl-4-sodium sulfonate)adamantane (FR-A) and 1,3,5-tri(phenyl-4-sodium sulfonate) adamantane (AS_3_) were able to pass a V-0 rating with 0.08 and 0.10 wt % loading, respectively [24,25]. However, the sulfur-based FRs, with the strong polarity of sulfonate, are easily soluble in water and are easily affected by dampness when the materials are in storage. Besides, the addition of FRs easily deteriorates the mechanical properties of materials, which is attributed to the poor interfacial interaction between FRs and the polymeric matrix, and causes some problems in processability [26].

To the best of our knowledge, nitrogen-based FRs are excellently water-resistant, while their flame retardant efficiency is barely satisfactory when used exclusively [27,28,29,30]. Meanwhile, in order to improve flame retardant efficiency and reduce the addition of flame retardant, combinations of various flame-retardant elements have been developed and widely reported recent years [31,32]. Additionally, adamantane and its derivatives are distinguished for their high rigidity, physical and chemical stability, and remarkable compatibility. Therefore, the transfer of the desired properties to the target polymer system has been an object of study [33,34,35].

In our study, 1,3,5,7-tetrakis(phenyl-4-sulfonyl-melamine)adamantine (ASN), containing sulfonate and triazine groups, was designed and synthesized with an adamantine moiety at the center. As expected, the rigidity, compatibility, and water-resistant properties of FRs could be enhanced, and it could be homogeneously dispersed to strengthen the interfacial interaction between FRs and the PC matrix. Afterwards, PC/ASN samples were prepared through thermal treatment, and pure PC and PC/KSS were also prepared as blank and control samples, respectively. Furthermore, the fire properties, water-resistance, and mechanical properties of PC composites were systematically investigated and revealed.

## 2. Materials and Methods

### 2.1. Materials

Polycarbonate 301-10 was provided by Dow Chemical Company (Midland, MI, USA) and dried for at least 8 h at 120 °C prior to compounding. Adamantane was sourced from Hangzhou Yang Li Petrochemical Co., Ltd. (Hangzhou, China). KSS employed as a reference FR was supplied by Guangdong Ever Sun Corp., (Dongguan, China). Aluminum chloride, chlorosulfonic acid, *t*-butyl bromide, and melamine were purchased from Aladdin Industrial Corporation (Shanghai, China) and used without further purification. 1,3,5,7-Tetrakis(phenyl-4-sulfonic)adamantane was prepared according to the published procedure [24].

### 2.2. Synthesis of ASN

**Scheme 1:**
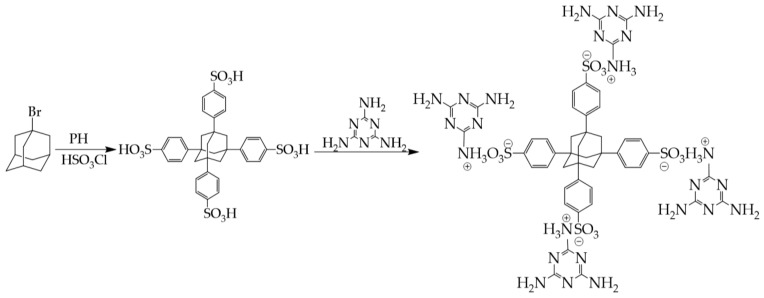
The synthetic route of 1,3,5,7-tetrakis(phenyl-4-sulfonyl-melamine)adamantine (ASN).

Melamine (3.65 g, 28.9 mmol) dissolved in deionized water (120 mL) was initially charged into a 500-mL three-neck flask equipped with reflux condenser and constant pressure funnel, followed by vigorously stirring at 85 °C until melamine was completely dissolved. Thereafter, 1,3,5,7-tetrakis(phenyl-4-sulfonic)adamantane (5.0 g, 6.6 mmol) dissolved in deionized water (50 mL) was added dropwise into the reaction system, followed by heating at 100 °C for 6 h. The reaction system was allowed to continually stir overnight, during which time it was cooled down to room temperature. After treatment, the dispersion was filtered, and the obtained residue was rinsed several times with deionized water. The final product was dried at reduced pressure at 80 °C for 12 h to give a white powder (7.3 g, 87.8% yield). The reaction formula is outlined in Scheme 1. Fourier transform infrared spectroscopy (FTIR) (KBr) *v*: 3350, 3157, 1659, 1396, 1164, 1023 cm^−1^. ^1^H NMR (DMSO-*d*_6_, 400 MHz): *δ* = 2.08 (s, 12H), 7.55–7.58 (m, 40H), 11.23 (s, 4H) ppm. Anal. calcd.: C, 43.66; H, 4.66; N, 26.57; S, 10.14. Found: C, 43.63; H, 4.67; N, 26.58; S, 10.12.

### 2.3. Preparation of PC/ASN Specimens

PC and ASN were dried in a vacuum oven at 120 °C for 12 h prior to processing. After treatment, PC and ASN were melt-mixed by an internal mixer (KY-3220-2L, Dongguan Houjie Machinery Equipment Factory, Dongguan, China) under 50 rpm at 250 °C for 10 min. The prepared composites were injection-molded into test pieces with standard sizes under compression (15 MPa) at 250 °C and then cooled down to room temperature to obtain PC/ASN specimens for further testing. In addition, pure PC and PC with KSS were also prepared by the same procedure for comparison. The formulations of a series of specimens are listed in Table 1.

### 2.4. Measurements and Characterization

Fourier transform infrared spectra were performed with a Thermo Electron Nicolet-6700 spectrometer (Thermo Nicolet Corporation, Waltham, MA, USA) at a spectrum resolution of 4 cm^−1^ with 16 scans over the range of 400–4000 cm^−1^. The hydrogen nuclear magnetic resonance (^1^H NMR) spectra were recorded on a Bruker AVANCE III 400-MHz superconducting spectrometer (Bruker Biospin AG, Fallanden, Switzerland) and the DMSO-*d*_6_ was used as solvent. Elemental analysis was obtained on a Perkin Elmer Series II 2400 elemental analyzer (Perkin Elmer Co., Ltd., Waltham, MA, USA).

Thermogravimetric analysis (TGA) was carried out with a NETZSCH STA 409PC thermal gravimetric analyzer (NETZSCH, Bobingen, Germany) under air or nitrogen atmosphere. The samples were placed in an aluminum crucible and ramped from room temperature up to 800 °C at a heating rate of 10 °C/min.

LOI tests were evaluated by using a DM-4022 oxygen index apparatus (Yangzhou Dongming Detection Instrument Technology Co., Ltd., Yangzhou, China) according to ASTM D2863-2008 standard procedure, and the dimensions of all specimens were 80 × 10 × 4 mm^3^. UL-94 vertical burning tests were measured by an AG5100A vertical-horizontal burning apparatus (Zhuhai Angui Test Equipment Co., Ltd., Zhuhai, China) in accordance with ASTM D3801 standard procedure. Each average result of LOI tests and UL-94 tests was obtained from six replicates.

Combustion behaviors of materials were measured by cone calorimeter tests based on the FTT 00007 cone calorimeter device (Fire Testing Technology Ltd., London, UK). Each sample, with three-dimensional size of 100 × 100 × 3 mm^3^, was wrapped in an aluminum foil and exposed horizontally to a radiant cone at a flux of 35 kW/m^2^ according to ISO 5660-1 standard procedure. The tests of each sample were replicated three times, and the average results were recorded.

For evaluation of the ability of water resistance, water soaking of the specimens was conducted at 70 °C for varying times, followed by drying at reduced pressure at 100 °C for 12 h. UL-94 tests were carried out to obtain the results of water-resistance.

Tensile tests and flexural tests of specimens were completed using a Zwick/Roell universal electronic tensile testing machine (Dongguan Puhua Experimental Equipment Co., Ltd., Dongguan, China) according to ISO-527-2:2012 and ISO-178:2003, respectively. Izod impact tests, according to ISO 180:2001, were evaluated by using Z5113 radial-boom impact tester (Guangzhou Xiangli Instrument Co., Ltd., Guangzhou, China). All data were the average of five independent measurements.

The SEM observation was performed on a Hitachi S-3400N scanning electron microscope (Hitachi, Tokyo, Japan) to investigate the microtopography of samples.

## 3. Results and Discussion

### 3.1. Characterization of ASN

The ASN centered on rigid adamantane was prepared in good yield via the synthetic route presented in Scheme 1. The chemical structure was well characterized through FTIR, ^1^H NMR, elemental analysis, and the thermostability was further confirmed by TGA.

**Figure 1:**
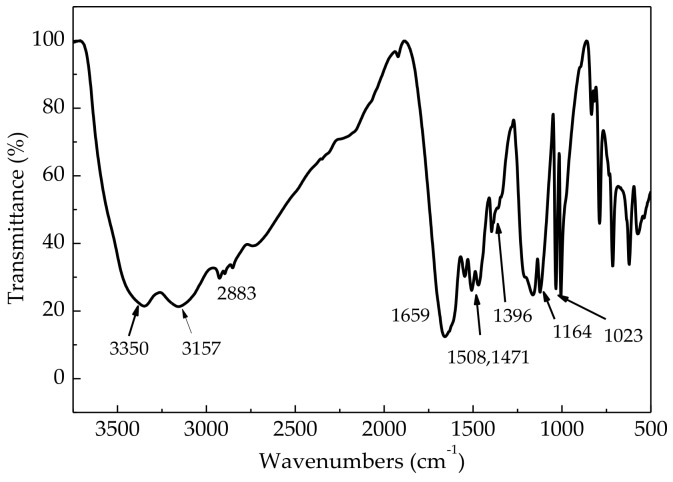
Fourier transform infrared (FTIR) spectrum of ASN.

FTIR analysis was used to obtain information regarding the chemical bonds of ASN, as shown in Figure 1. The broad IR bands around 3350 and 1659 cm^−1^ should be attributed to the stretching vibration and scissoring vibration of N–H bond in melamine groups, respectively. Three absorptions were detected at 1508, 1471, and 1396 cm^−1^ corresponding to the stretching vibrations of the triazine ring [36]. It is noteworthy that the absorption of –NH_3_^+^ appears at 3157 cm^−1^ [37]. Moreover, the absorptions at 1164 and 1023 cm^−1^ are ascribed to the asymmetric and symmetric stretching vibrations of the –S=O group, respectively [25].

**Figure 2:**
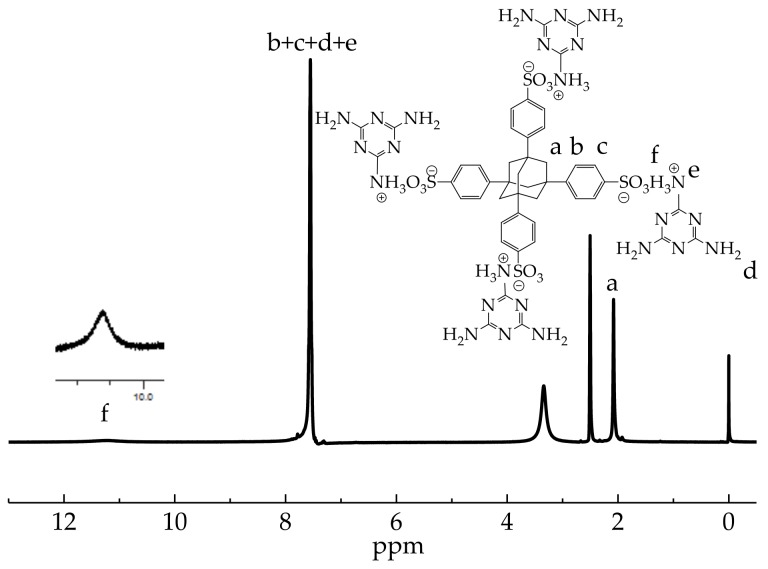
^1^H NMR spectrum of ASN.

As shown in Figure 2, ^1^H NMR spectroscopy was further carried out to clarify the structure of ASN. The chemical shifts at 7.55–7.58 ppm (m, 40H) are assigned to the aromatic protons and melamine protons (labeled b–e). The weak signals emitted by –SO_3_H (labeled f) locate at 11.23 ppm (s, 4H). Additionally, the strong resonance signals at 2.08 ppm (s, 12H) arise from the CH_2_ protons (labeled a). Conclusively, on the basis of the analysis of FTIR and ^1^H NMR spectra above, it is believed that ASN was synthesized successfully.

**Figure 3:**
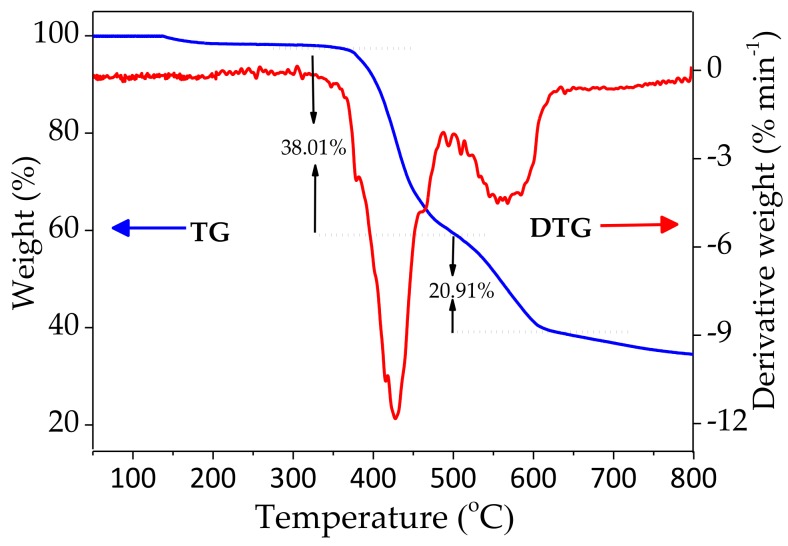
Thermogravimetric (TG) and derivative thermogravimetric (DTG) curves of ASN under N_2_ atmosphere.

Thermal properties of FRs are crucial to their application. Thus, the thermal stability of ASN was studied by TGA under nitrogen, and the thermogravimetric (TG) and derivative thermogravimetric (DTG) curves are presented in Figure 3. The thermal degradation of ASN displayed a two-stage pattern, and the 5% weight loss temperature (*T*_5%_) of ASN was 385 °C. According to the weight loss percent on TG curves, the weight loss between 345 and 496 °C should be attributed to the decomposition of melamine, while the weight loss between 496 and 635 °C should correspond to the released SO_2_ from the pyrolysis of sulfonate groups. Proper decomposition temperature is particularly vital to additive-type FRs. The *T*_5%_ of ASN is much higher than the thermo-processing temperature of PC composites (250 °C). Namely, the prepared ASN can satisfy the demands for FRs of PC with respect to thermal stability. Notably, ASN has a residue of 36.8% at 800 °C and it can be revealed that ASN predominantly decomposes to form volatile products.

### 3.2. Thermal Degradation of PC Samples

**Figure 4:**
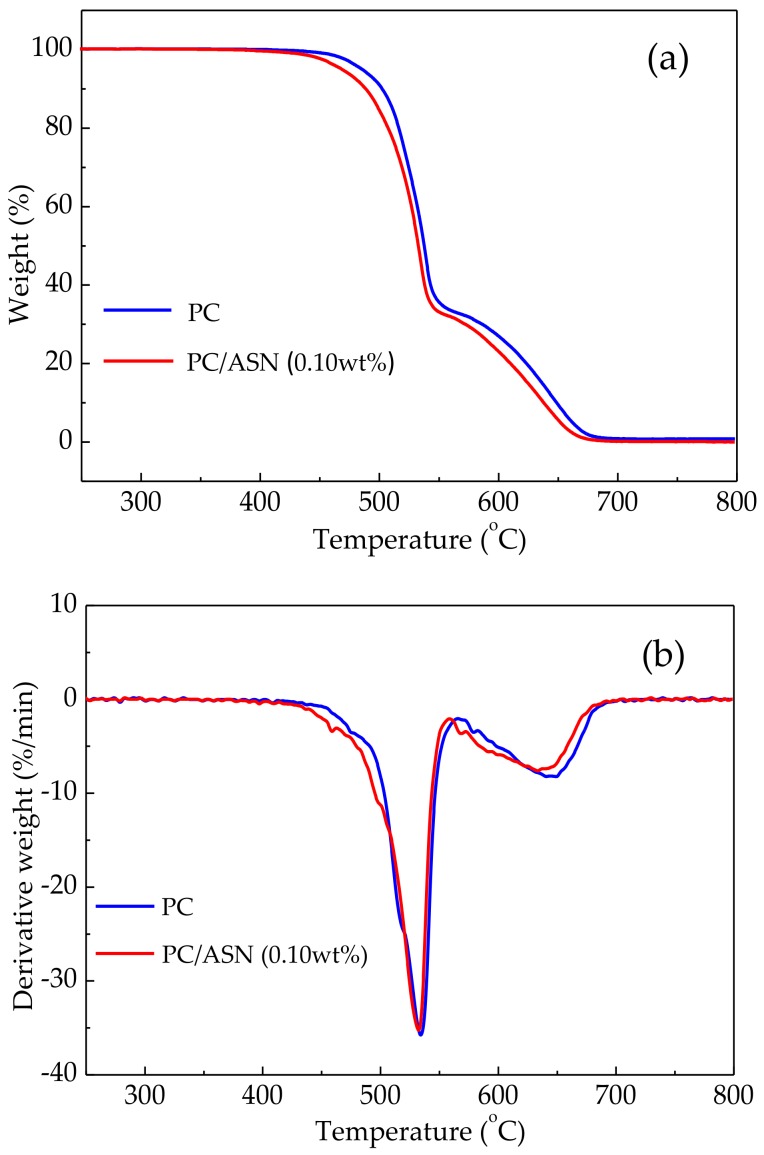
(**a**) TG and (**b**) DTG curves of PC and PC/ASN (0.10 wt %) under air atmosphere.

The thermostability and thermal degradation of PC/ASN composites were investigated by TGA under air, and the results obtained are given in Figure 4. Overall, TG curves of both pure PC and PC/ASN (0.10 wt %) show a two-step decomposition. Interestingly, the PC/ASN (0.10 wt %) degraded before pure PC. *T*_5%_ of PC/ASN (0.10 wt %) and pure PC were 467 and 485 °C, respectively. Besides, a similar phenomenon was observed in the DTG curves of pure PC and PC/ASN (0.10 wt %), presented in Figure 4b. The thermal degradation process of pure PC displayed two main weight-loss stages in which the temperatures of maximum weight loss rate (*T*_max_) were found at 534 and 649 °C, respectively. However, the *T*_max_ of PC/ASN (0.10 wt %) were found at 532 and 633 °C, respectively. *T*_5%_ of PC/ASN (0.10 wt %) was observed at an unnoticeably lower temperature (reduced by 18 °C), which is attributed to the slight addition of ASN (0.10 wt %) and whose sulfonate groups accelerated the decomposition of PC and the formation of residual char [22,38].

### 3.3. Flammability Characteristics

#### 3.3.1. LOI and UL-94 Tests

The fire performance of PC/ASN composites as characterized by the LOI and UL-94 tests was anticipated to be improved, and the correlative results are summarized in Table 1. In detail, the LOI value of PC/ANS (0.06 wt %) was 28.5%, which was slightly superior to pure PC, while the UL-94 V-0 rating was not passed. Furthermore, PC/ASN (0.10 wt %) exhibited striking potency in retarding PC with a V-0 rating and an LOI value of 30.1%. With further increasing addition of ASN, the fire performance showed no improvement in LOI value, while the UL-94 V-0 rating was successfully maintained. By comparison, the control specimens loaded with 0.10 wt % KSS could only reach a UL-94 V-2 rating and a LOI value of 28.6%. According to the analyses above, the fire properties of PC were significantly improved with a minor amount of ASN loaded. It is conjectured that this interesting phenomenon is attributable to the high flame-retardant element content of ASN, such as 10.3% sulfur and 26.9% nitrogen. It is worth noting that compared with PC/ASN (0.10 wt %), PC/FR-A (0.06 wt %) only exhibited a UL-94 V-1 rating and an LOI value of 24.0% in the same sulfur content (0.01% for S in both PC composites) [24]. Hence, the presence of the triazine group may have had an enhancement effect for the sulfonate flame retardants.

#### 3.3.2. Cone Calorimeter Tests

**Figure 5:**
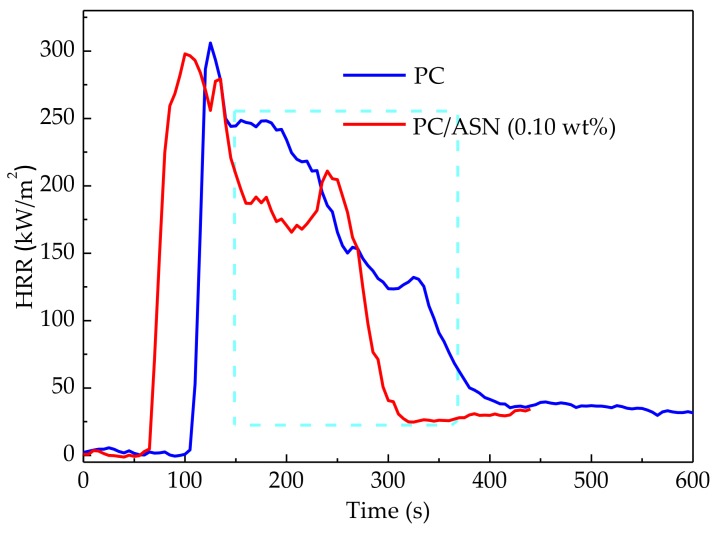
Heat release rate curves of PC and PC/ASN (0.10 wt %).

**Figure 6:**
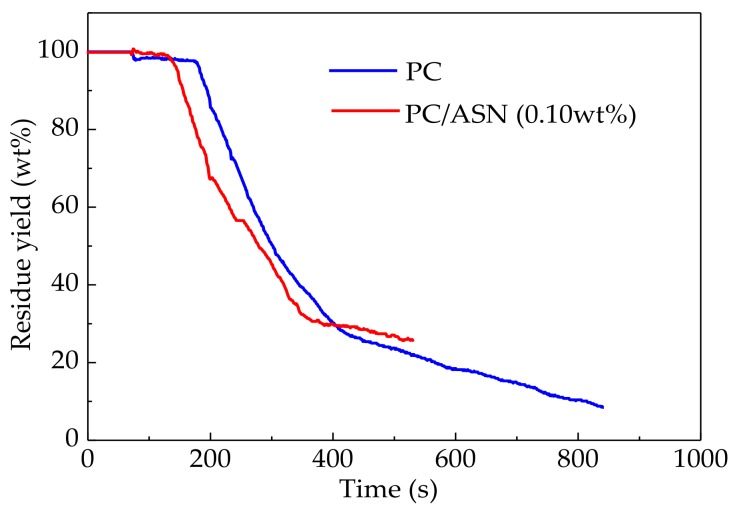
Residue yield curves of PC and PC/ASN (0.10 wt %).

**Figure 7:**
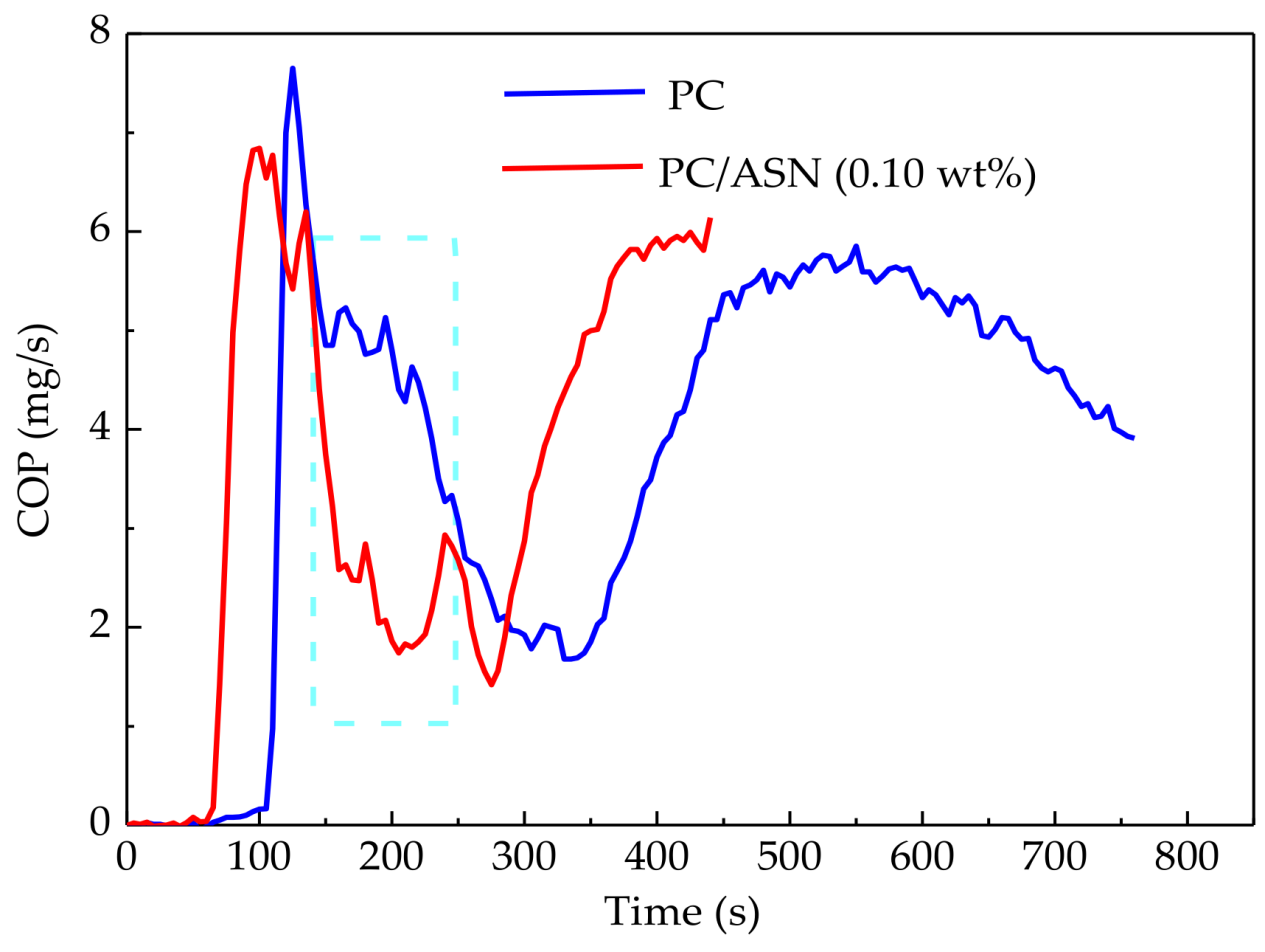
CO release rate curves of PC and PC/ASN (0.10 wt %).

Cone calorimeter tests bring quantitative analysis to material flammability research by evaluating various parameters shown in Table 2, including time to ignition (TTI), peak of heat release rate (pk-HRR), mean heat release rate (m-HRR), total heat release (THR), effective heat combustion (EHC), residue yield (RY), specific extinction area (SEA), and peak of smoke production rate (pk-SPR). In addition, the HRR, residue yield, and CO release rate for pure PC and PC/ASN (0.10 wt %) are illustrated in Figure 5, Figure 6 and Figure 7, respectively.

**Figure 8:**
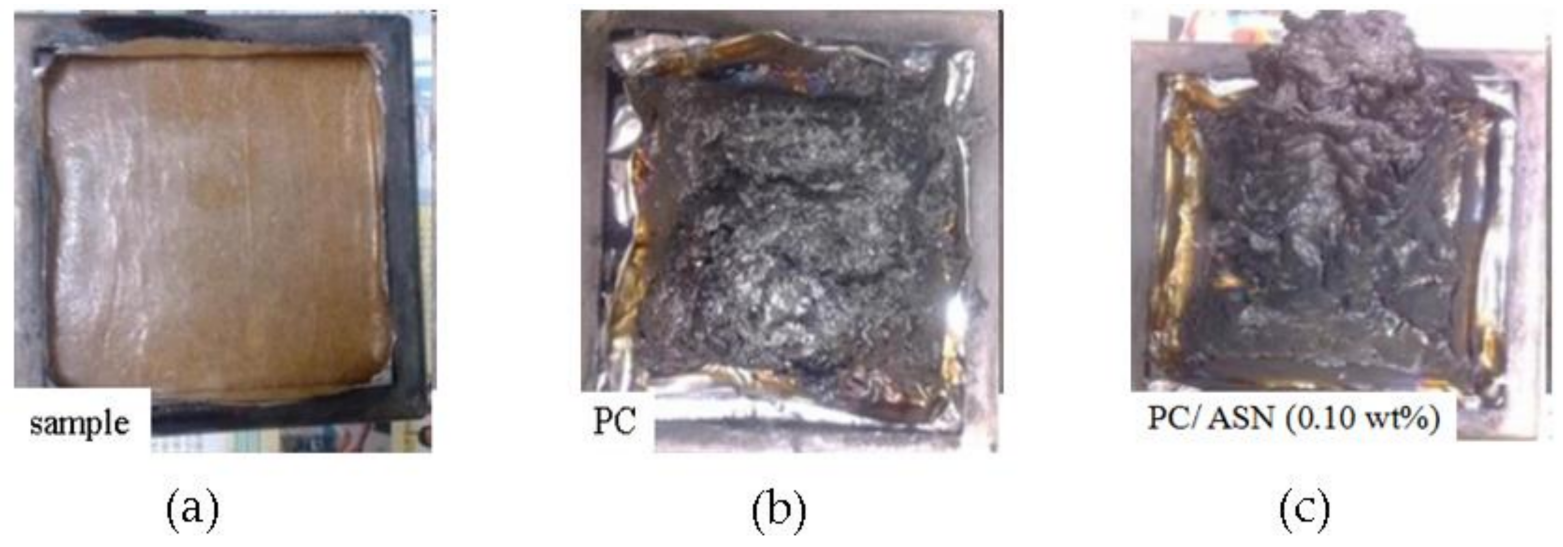
Digital photos of (**a**) the sample before cone calorimeter tests, and the residues after cone calorimeter tests of (**b**) PC and (**c**) PC/ASN (0.10 wt %).

HRR has been believed to be one of the most important parameters for the evaluation of the fire safety of materials [14]. As shown in Figure 5, HRR values of pure PC sharply increased to a maximum value (pk-HRR) of 305.9 kW/m^2^ at 125 s. By comparison, pk-HRR slightly decreased to 297.8 kW/m^2^ for PC/ASN (0.10 wt %). Distinctly, the HRR curve of PC/ASN (0.10 wt %) dropped dramatically at the time range of 150–360 s. From Table 2, the THR of PC/ASN (0.10 wt %) decreased from 61.0 to 49.3 MJ/m^2^ compared with that of pure PC. It is speculated that the protective layers of PC blended ASN were formed earlier on the surface, and the layers served as a thermal insulation layer to inhibit the pyrolysis of PC composites [39]. Moreover, the TTI of pure PC was 102 s, while it was decreased to 60 s with the addition of 0.10 wt % ASN. It can also be noted from Figure 6 that the composites containing 0.10 wt % ASN lost weight in advance and the residual yields of PC and PC/ASN (0.10 wt %) were 8.5% and 25.7% (including the unburned PC encased in char residual), respectively. It is deduced that the ASN accelerated the decomposition of PC in advance and quickly facilitated the formation of residual char to impede the further decomposition of PC composites. EHC, defined as the ratio of m-HRR to the average mass loss rate, is an important parameter to disclose the burning degree of volatile species generated from materials during combustion [40]. As shown in Table 2, the EHC of PC/ASN (0.10 wt %) showed little change compared with that of pure PC (slightly increased by 6%). Combining with the residue yield of PC and PC/ASN (0.10 wt %), which is respectively 8.5 and 25.7 wt %, it is suggested that the flame-retardant effect of ASN mainly functions in the condensed phase [41]. Besides, the major products of the inefficient combustion of materials include carbon monoxide [42]. In this study, the mean CO production of PC/ASN (0.10 wt %) greatly decreased by 50.7% relative to that of pure PC, which will provide a relative safer situation for people in a fire hazard. Regarding the parameters representing the generation of smoke, the pk-SPR of PC/ASN (0.10 wt %) decreased from 0.133 to 0.113 m^3^/s. Nevertheless, SEA rose by 4.7% compared to that of pure PC, which signifies that the introduction of ASN will give rise to a slight increase of smoke. Meanwhile, a similar phenomenon was not noticed from the cone calorimeter test results of FR-A or AS_3_. It is well-known that triazine-containing flame retardants function in the gas phase [43]. Being associated with the relatively loose residual char of PC/ASN (0.10 wt %) shown in Figure 8, it is conjectured that the slight increase of SEA is attributable to the noncombustible gas released from the pyrolysis of the triazine group, and the gas will also blow aloft some solid particles generated from the burning of the PC matrix.

### 3.4. Analysis of Residual Char

**Figure 9:**
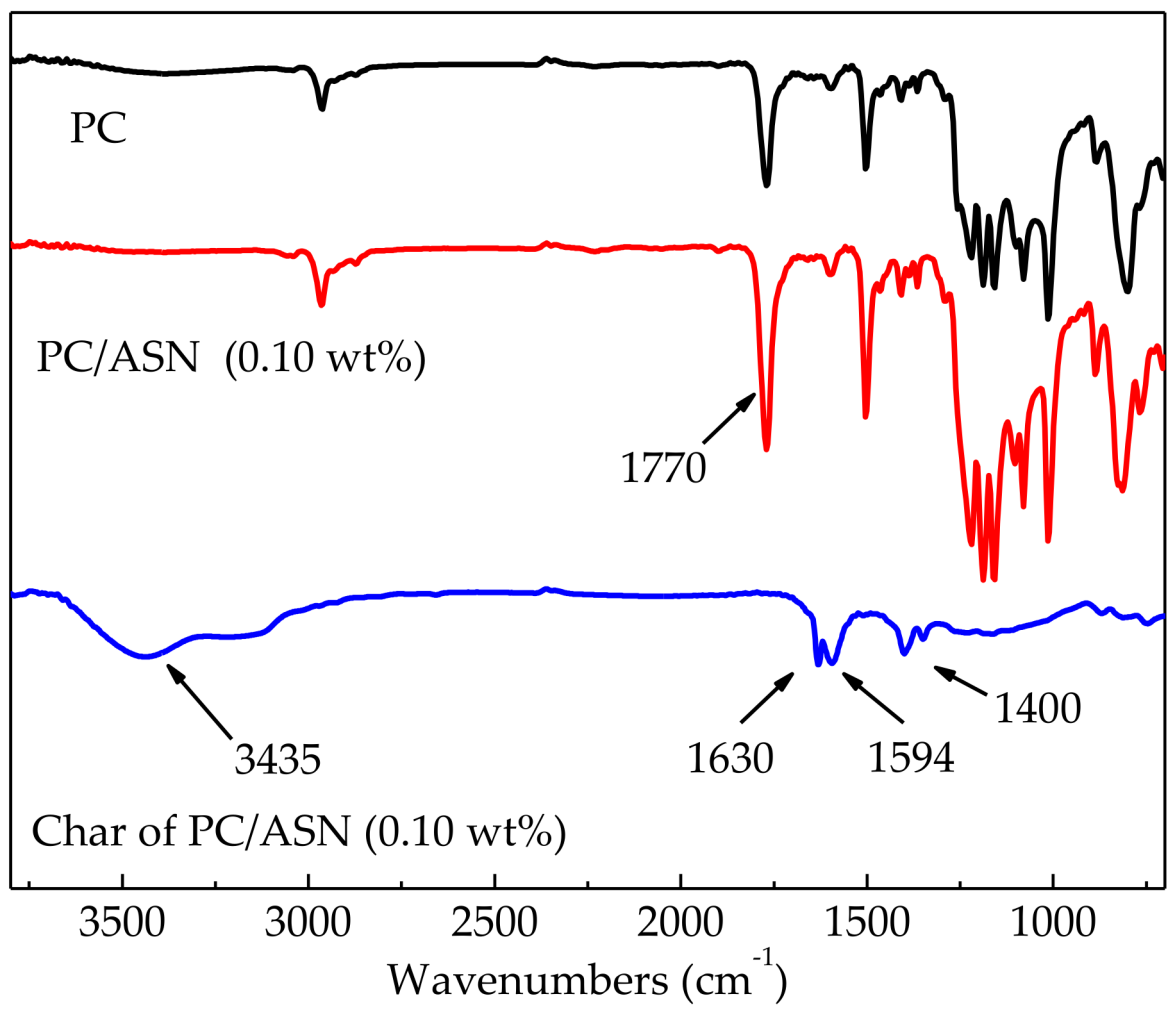
FTIR spectrum of PC, PC/ASN (0.10 wt %), and the char of PC/ASN (0.10 wt %).

To further investigate the char formation of PC/ASN composites, charred residue of PC/ASN (0.10 wt %) produced by UL-94 tests was analyzed by FTIR. The characteristic bonds on the spectrum are ascribed to corresponding structure, as shown in Figure 9. The FTIR curves of pure PC and PC/ASN (0.10 wt %) were basically identical, and such a phenomenon is ascribed to the trace amounts of ASN loaded in the materials. With regard to the FTIR curve of residual char, the stretching and bending vibrations of the –OH group were observed at 3435 and 1400 cm^−1^, respectively. In addition, the absorptions of C=C from aromatic nucleus appeared at 1630 and 1594 cm^−1^ [25]. Melamine was not observed in residue, evidencing that it was released to the gas phase. From the FTIR results above, it is extrapolated that some compounds containing aromatic alcohol were generated in the pyrolysis of PC and further crosslinked automatically to develop a carbon layer. Therefore, the unburned PC was protected and further decomposition was prevented, which coincides with the residue yield of PC/ASN (0.10 wt %) and pure PC (25.7% and 8.5%, respectively).

### 3.5. Water Resistance

To evaluate the water resistance of PC samples, water soaking of the testing specimens was conducted for varying periods of time at 70 °C, and the corresponding UL-94 tests were revalued (Table 3). In detail, the flame retardant properties of PC/FR-A and PC/KSS were severely impaired after water soaking. In contrast, PC/ASN samples were less affected and maintained the V-0 rating. Consequently, it is validated that PC/ASN composites are highly water resistant and have an application prospect in PC outdoor applications.

### 3.6. Mechanical Properties

**Figure 10:**
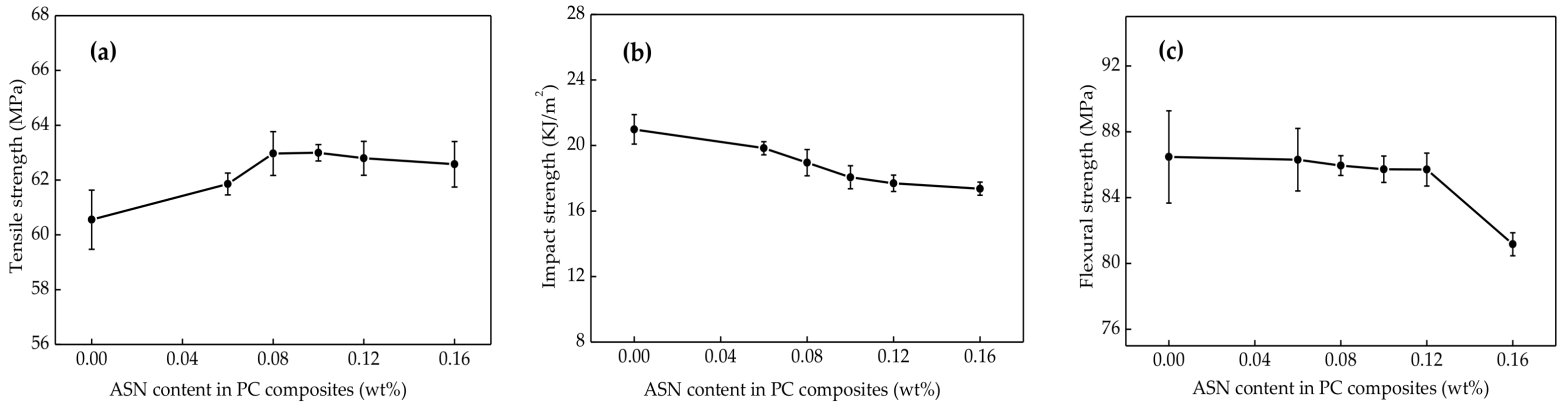
Mechanical properties of PC/ASN samples: (**a**) tensile strength; (**b**) impact strength; (**c**) flexural strength.

The effects of ASN loading on tensile strength, impact strength, and flexural properties are presented in Figure 10. As expected, with the addition of ASN, the curve of tensile strength showed an upgrade tendency under investigation. Typically, the tensile strength reached a maximum value (63.00 MPa) when 0.10 wt % ASN was blended into PC, and increased by 4.0% compared with that of pure PC (60.55 MPa). A further increase in ASN loading resulted in a slight decline in tensile strength. A certain amount of added ASN not only led to no deterioration, but also provided slight improvement in tensile properties. Furthermore, with a low amount of ASN added (i.e., less than 0.12 wt %), the flexural strength of the samples studied was barely changed. However, there was a sharp decrease in the flexural strength once the loading of ASN was increased to 0.16 wt %. In contrast, PC/KSS (0.08 wt %) and PC/KSS (0.10 wt %) had the tensile strengths 59.88 and 60.1 MPa, and had flexural strengths 76.88 and 78.88 MPa, respectively [25]. These were both lower than that of pure PC. Besides, the curve of notch impact strength indicated a continuously declining trend with the addition of ASN. Under the optimal content of ASN (0.10 wt %), the notch impact strength decreased by 13.7% from 20.92 to 18.06 KJ/m^2^. The notch impact strength of PC samples is apparently sensitive to the additives [44], and the deterioration of the notch impact strength for composites with the addition of FRs has been reported by some researchers [45,46,47]. For example, the notch impact strength of PC specimens was reduced by more than 100% with the optimum additive amount of hexakis(4-nitrophenoxy) cyclotriphosphazene (HNTP, 6 wt %) [46] and tris(phenoxy)trifluorocyclotriphosphazenes (TCTP, 6 wt %) [47]. Consequently, the novel flame retardant ASN can effectively alleviate the deterioration of the mechanical properties of polymeric matrix, and even improve the tensile strength to a certain extent in some cases.

**Figure 11:**
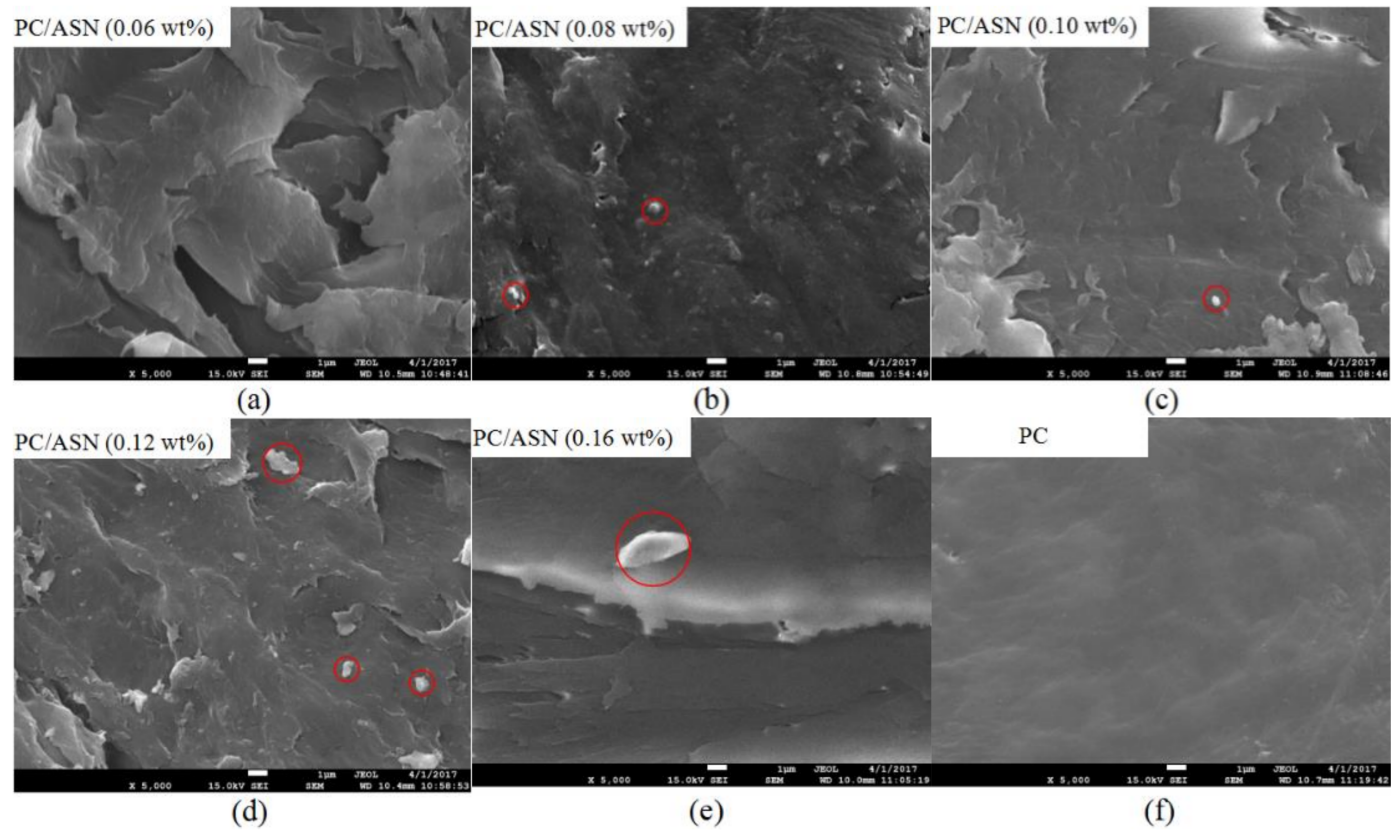
SEM micrographs of PC and PC/ASN: (**a**) PC/ASN (0.06 wt %); (**b**) PC/ASN (0.08 wt %); (**c**) PC/ASN (0.10 wt %); (**d**) PC/ASN (0.12 wt %); (**e**) PC/ASN (0.16 wt %); (**f**) pure PC.

The mechanical properties are cohesively associated with the microstructures of composites. To investigate the mechanism for the effects of ASN loading on the mechanical properties of PC samples, SEM measurements were carried out. From Figure 11a–d, it is clear that ASN particles were generally well-dispersed in the PC matrix, which reveals remarkable compatibility of ASN with PC on account of the introduction of the adamantane group. Thus, it is safe to conjecture that the improvement of tensile strength is attributable to the large interfacial area and strong interaction between PC matrix and ASN. However, more than 0.16 wt % ASN loading resulted in partial aggregation of ASN particles, which served as stress concentrators, leading to a slight decline in the tensile strength and a sharp decrease in flexural strength [48]. Furthermore, it is inevitable that a very tiny amount of white spots are still noticed from SEM micrographs (circled in red). Hence, the notch impact strength showed a declining trend in our work because of its sensitivity to the additives.

## 4. Conclusions

A novel S/N-based synergistic flame retardant (ASN) with favorable water-resistance was successfully synthesized and used to flame retard PC. With the incorporation of ASN, flame-retardant properties of PC samples were greatly improved. For example, PC/ASN (0.10 wt %) had a V-0 rating and an LOI value of 30.1%, although its thermostability was slightly inferior to pure PC to some extent. In addition, ASN had an active role in restraining heat release and reducing poison gas release. PC with incorporated ASN also showed excellent water-resistance, so it is able to meet the more stringent requirements of certain applications, such as outdoor PC applications. In addition, with the analyses of mechanical properties, although the notch impact strength of PC/ASN blends was weakened to a certain degree, the tensile strength and rigidity of specimens were improved with the addition of FR-ASN. Substantially building on this pioneering work, it is believed that ASN is an efficient flame-retardant for PC, with a huge prospect for application.

## Figures and Tables

**Table 1:** Formulations of polycarbonate (PC) samples and the results of UL-94 and limited oxygen index (LOI) tests. KSS: potassium diphenylsulfone sulfonate.

Samples	PC/wt %	ASN/wt %	KSS/wt %	Dripping	Rating	LOI/%
PC	100.00	0.00	0.00	Yes	V-2	25.8
PC/ASN (0.06 wt %)	99.94	0.06	0.00	No	V-1	28.5
PC/ASN (0.08 wt %)	99.92	0.08	0.00	No	V-1	29.7
PC/ASN (0.10 wt %)	99.90	0.10	0.00	No	V-0	30.1
PC/ASN (0.12 wt %)	99.88	0.12	0.00	No	V-0	30.5
PC/ASN (0.16 wt %)	99.84	0.16	0.00	No	V-0	30.2
PC/KSS (0.10 wt %)	99.90	0.00	0.10	Yes	V-2	28.6

**Table 2:** Cone calorimeter tests results of PC and PC/ASN (0.10 wt %). EHC: effective heat combustion; m-HRR: mean heat release rate; pk-HRR: peak of heat release rate; pk-SPR: peak of smoke produce rate; RY: residue yield; SEA: specific extinction area; THR: total heat release; TTI: time to ignition.

Parameters	PC	PC/ASN (0.10 wt %)
TTI (s)	102	60
pk-HRR (kW/m^2^)	305.9	297.8
m-HRR (kW/m^2^)	147.3	129.5
THR (MJ/m^2^)	61.0	49.3
EHC (MJ/kg)	19.7	21.0
RY (wt %)	8.5	25.7
SEA (m^3^/kg)	680.5	712.5
pk-SPR (m^3^/s)	0.133	0.113
mean CO production (kg/kg)	0.148	0.073

**Table 3:** UL-94 results of PC samples after water treatment with different times. FR-A: 1,3,5,7-tetrakis(phenyl-4-sodium sulfonate)adamantane.

Samples	PC/ASN (0.10 wt %)	PC/FR-A (0.10 wt %)	PC/KSS (0.10 wt %)
0 h	V-0	V-0	V-1
24 h	V-0	V-1	V-2
48 h	V-0	V-1	V-2
72 h	V-0	V-2	V-2
